# IL‐8 and CXCR1 expression is associated with cancer stem cell‐like properties of clear cell renal cancer

**DOI:** 10.1002/path.5267

**Published:** 2019-04-11

**Authors:** Claudia Corrò, Marc E Healy, Stefanie Engler, Bernd Bodenmiller, Zhe Li, Peter Schraml, Achim Weber, Ian J Frew, Markus Rechsteiner, Holger Moch

**Affiliations:** ^1^ Department of Pathology and Molecular Pathology University Hospital Zurich Zurich Switzerland; ^2^ Life Science Zurich Graduate School ETH and University of Zurich Zurich Switzerland; ^3^ Institute of Molecular Life Sciences University of Zurich Zurich Switzerland; ^4^ Division of Genetics, Department of Medicine Brigham and Women's Hospital and Harvard Medical School Boston MA USA; ^5^ Clinic of Internal Medicine I Faculty of Medicine, Medical Center – University of Freiburg Freiburg im Breisgau Germany

**Keywords:** IL‐8, CXCR1, CXCL8, stem cells, renal cancer, xenografts, metastasis

## Abstract

Recent studies suggest that clear cell renal cell carcinoma (ccRCC) possesses a rare population of cancer stem cells (CSCs) that might contribute to tumor heterogeneity, metastasis and therapeutic resistance. Nevertheless, their relevance for renal cancer is still unclear. In this study, we successfully isolated CSCs from established human ccRCC cell lines. CSCs displayed high expression of the chemokine IL‐8 and its receptor CXCR1. While recombinant IL‐8 significantly increased CSC number and properties *in vitro*, CXCR1 inhibition using an anti‐CXCR1 antibody or repertaxin significantly reduced these features. After injection into immune‐deficient mice, CSCs formed primary tumors that metastasized to the lung and liver. All xenografted tumors in mice expressed high levels of IL‐8 and CXCR1. Furthermore, IL‐8/CXCR1 expression significantly correlated with decreased overall survival in ccRCC patients. These results suggest that the IL‐8/CXCR1 phenotype is associated with CSC‐like properties in renal cancer. © 2019 The Authors. *The Journal of Pathology* published by John Wiley & Sons Ltd on behalf of Pathological Society of Great Britain and Ireland.

## Introduction

Renal cell carcinoma (RCC) is a malignant neoplasm derived from renal tubular cells and affects approximately 64 000 people worldwide every year 1. Clear cell RCC (ccRCC) is the most common subtype of RCC, which is characterized by genetic and morphologic intratumor heterogeneity combined with poor response to radiotherapy and chemotherapy 2, 3, 4.

Recently, it has been hypothesized that cancer stem cells (CSCs) might contribute to tumor heterogeneity, metastasis and resistance to therapy in renal cancer 5, 6, 7, 8, 9. Numerous studies have since tried to isolate CSCs from RCC 10, 11, 12, 13, 14, with CD105, ALDH1, OCT4, CD133, and CXCR4 reported as markers of cancer stem‐like cells from RCC 5, 11, 15, 16, 17, 18, 19. However, contrasting results have been reported in the literature on the use of these biomarkers 18, 20, 21, 22, 23.

In search of novel renal CSC markers, we identify an essential role for IL‐8 (CXCL8)/CXCR1 signaling in proliferation, migration, invasion, sphere formation and self‐renewal capabilities of ccRCC‐derived tumor cells, suggesting a stemness signature of this cell population. Furthermore, we highlight the therapeutic potential of blocking IL‐8/CXCR1 signaling in ccRCC.

## Materials and methods

### Ethics statement

The local ethics commission for (KEK‐ZH‐Nr. 2011‐0072 and KEK‐ZH‐Nr. 2014‐0614) approved the use of human material for this study. Detailed information regarding the cohort description are provided in supplementary material, Supplementary materials and methods.

NOD.Cg‐Prkdcscid Il2rgtm1Wjl/SzJ (NSG) mice were purchased from The Jackson Laboratory (Mouse strain #005557). Housing of animals and all experimental procedures were performed at the University Hospital Zurich in accordance with the Cantonal Veterinary Office (Zurich, Switzerland) under the license number ZH104/2015 (see supplementary material, Supplementary material and methods).

### Immunohistochemistry

Tissue and cell culture microarrays (T/CMAs) were constructed as described previously 24. Antibodies used for IHC are listed in supplementary material, Table S1. Quantification of staining intensities was performed using Fiji 25.

### Cell lines

Details of the cell lines used are provided in supplementary material, Supplementary materials and methods.

### Western blotting

Western blotting was performed as described previously 26. The following antibodies were employed: anti‐human CXCR1 (clone 42705, 5 μg/μl, R&D Systems, Minneapolis, MN, USA), anti‐human IL‐8 (clone 6217, 10 μg/μl, R&D Systems), HIF1α (1:1000, Novus Biologicals, Littleton, CO, USA), HIF2α (1:500, Abnova, Jhongli, Taiwan), CAIX (clone M75, 1:1000, J. Zavada, Prague, Czech Republic) and β‐actin (1:2000, Chemicon International, Temecula, CA, USA).

### Cell invasion and migration

Details for cell invasion and migration assays are provided in supplementary material, Supplementary materials and methods.

### Hypoxia

Details for hypoxia experiments are provided in supplementary material, Supplementary materials and methods.

### Tumorsphere culture

Details for tumorsphere culture are provided in supplementary material, Supplementary materials and methods.

### Sphere formation assay

Tumor cells growing in adherent conditions were transferred into six‐well ultra‐low attachment plates containing tumor sphere medium at 5 × 10^3^ cells/well similarly to the generation of tumor sphere cultures (see supplementary material, Supplementary material and methods). Spheres were grown for 15 days. In enrichment steps, spheres were split and plated at the same initial cell density of 5 × 10^3^ cells/well every 15 days and counted at the end of each period. This step was repeated at least five times. In this manuscript, we always refer to CSCs enriched in the sphere formation assay for at least three passages.

### Limiting dilution assay

Details for limiting dilution assay are provided in supplementary material, Supplementary materials and methods.

### Clonogenic assay

Details for clonogenic assay are provided in supplementary material, Supplementary materials and methods.

### Quantitative reverse transcription polymerase chain reaction

Details for RT‐qPCR are provided in supplementary material, Supplementary materials and methods.

### Enzyme‐linked immunosorbent assay

Details for ELISA are provided in supplementary material, Supplementary materials and methods.

### Human cytokine array

Details for human cytokine array are provided in supplementary material, Supplementary materials and methods.

### Xenografts

Female NSG mice were subcutaneously injected with parental or sphere‐derived cells from the ccRCC cell lines 769P and Caki‐1. 10^6^ cells, 10^4^ cells or 10^2^ cells were injected into the left flank of each mouse in 50% mixture of Matrigel (Growth Factor Reduced 35623, Corning, Corning, NY, USA) and PBS in triplicates. Each group of three mice received either tumor cells or only matrigel and PBS. Mice were monitored daily in the first 10 days post‐injection and when the tumor started to grow, otherwise every second day. Tumor size was measured using a caliper. When the tumor size reached 1 cm^3^, mice were euthanized and the tumor was harvested. Mice were extensively inspected in order to determine the presence of macrometastasis.

### Flow cytometry

Flow cytometry was performed on the LSR Fortessa instrument (BD Biosciences, San Jose, CA, USA) at the Flow cytometry Facility (University of Zurich, Switzerland). The ability to discriminate the side population (SP) is based on the differential efflux of Hoechst 33342 by a multidrug resistance transporter. Stem cells typically possess higher activity and/or exhibit higher expression levels of the multidrug transporters. SP stands out as the portion of cells able to extrude the dye against a concentration gradient when compared to cells not having stem cell features. Therefore, SP was identified as the portion of events that disappeared upon treatment with 100 μm verapamil which blocks the efflux of Hoechst in the CSCs (see supplementary material, Supplementary materials and methods).

### Mass cytometry

Mass cytometry (cyTOF) uses rare earth metal as reporters linked to antibodies for mass cytometry to provide high‐throughput identification and quantification of markers expressed in individual cells 27. This analysis was performed as described in supplementary material, Supplementary material and methods.

### Drug treatment

Different compounds were used in the treatment of cells in this study. Repertaxin (HY15251, Hycultec, Beutelsbach, Germany) is a small molecule with potent inhibitory activity on CXCR1/2. It was used at 100 nm concentration. Anti‐CXCR1 antibody (clone 42705, R&D Systems) was used at the concentration of 20 μg/ml, whereas to achieve CXCR1 activation the human recombinant IL‐8 (208‐IL, R&D Systems) was used at 1 μg/ml.

### Statistical analyses

Analysis between groups was carried out with ANOVA and Bonferroni multiple comparison. Student's *t‐*test, Kruskal–Wallis test associated with Dunn's multiple comparison, and Wilcoxon's signed rank test were also employed. Survival curves were estimated with the Kaplan–Meier method and the log‐rank test. All these analyses were performed using GraphPad Prism 5 (GraphPad Software Inc., San Diego, CA, USA). *P* values <0.05 were considered statistically significant and presented as follows: ^*^ *p* < 0.05; ^**^ *p* < 0.02; ^***^ *p* < 0.001; ^****^ *p* < 0.0001. With *P* value >0.05, results were considered nonsignificant (n.s.).

## Results

### ccRCC contains CSC populations capable of self‐renewal

**Figure 1:**
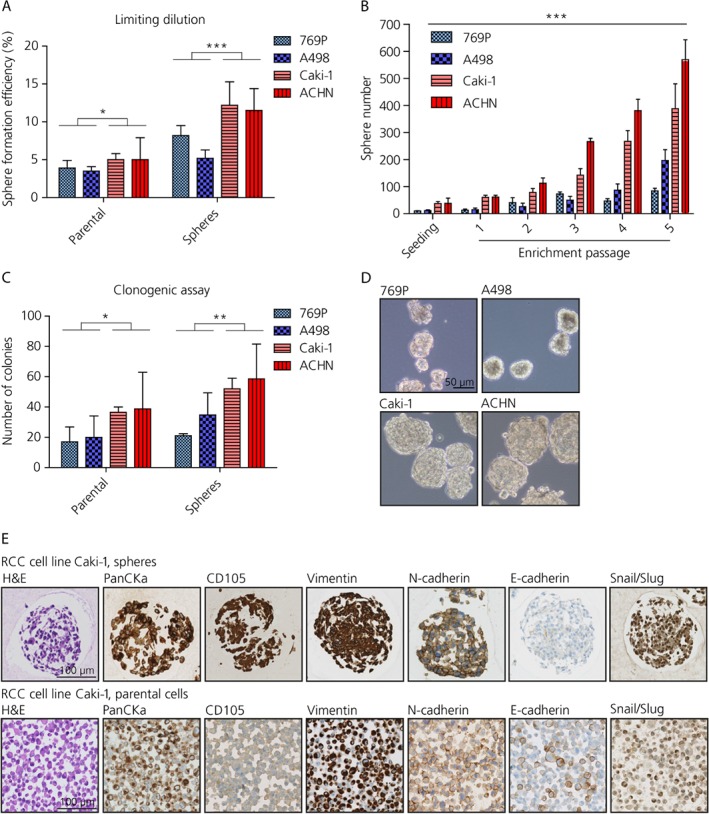
ccRCC cell lines possess a population of cancer cells with stem‐like features. (A) Limiting dilution assay displaying sphere formation capability in ccRCC cell lines (unpaired *t*‐test, *n* = 5). (B) Self‐renewal properties assessed by the sphere formation assay (two‐way ANOVA, *n* = 3). (C) Self‐renewal properties evaluated in the clonogenic assay (unpaired *t*‐test, *n* = 3). (D) Representative images of spheres derived from ccRCC cell lines. Scale bar: 50 μm. (E) Side‐by‐side immunohistochemical stains for Caki‐1 (spheres: upper panels; parental cells: lower panels). Scale bar: 100 μm.

CSCs were isolated from four ccRCC cell lines (769P, A498, Caki‐1 and ACHN) by sphere formation assay. Metastasis‐derived cultures (Caki‐1 and ACHN) showed a more pronounced sphere formation capability, which ranged between 1.2 and 3.5% spheres formed, compared to primary tumor‐derived cultures (769P and A498) that ranged between 0.5 and 0.6% (Table 1). Supportive evidence from limiting dilution assays suggests an increased CSC fraction in the metastatic sites compared to the primary tumors (*P* values 0.039 and 0.0005, respectively; Figure 1A).

**Table 1:** Sphere formation efficiency in primary tumor‐ and metastasis‐derived ccRCC cell lines

ccRCC cell line	769P	A498	Caki‐1	ACHN
Isolation site	Primary tumor	Primary tumor	Metastasis	Metastasis
Spheres formed*	10.2 ± 0.7	12.4 ± 3.0	23.4 ± 2.2	69.8 ± 19.2
Efficiency	0.5%	0.6%	1.2%	3.5%

* Spheres formed/2000 cells seeded.

Self‐renewal properties were evaluated by enriching the CSC population in the sphere formation assay over five passages. Caki‐1 and ACHN showed not only significantly greater sphere formation capability but also self‐renewal features when compared to 769P and A498 (*p* < 0.001; Figure 1B). Enhanced clonogenic activity when plated under adherent conditions at low density was observed for Caki‐1 and ACHN compared to 769P and A498, suggestive of increased self‐renewal properties (*P* values 0.041 and 0.006, respectively; Figure 1C and see supplementary material, Figure S1A). In addition, spheres derived from Caki‐1 and ACHN were bigger in size than the spheres formed by 769P and A498, ranging between 20 and 300 μm (Figure 1D).

Increased expression of EMT markers such as vimentin, Snail/Slug and N‐cadherin, and the CSC marker CD105 was found by IHC in the spheres derived from Caki‐1 compared to the corresponding adherent cells, whereas a decreased expression of E‐cadherin was observed (Figure 1E). Similarly, 769P, A498, and ACHN showed EMT (data not shown).

The capability to revert the EMT phenotype was also investigated by seeding spheres onto normal adherence tissue culture dishes. Spheres derived from Caki‐1 were able to attach again to the surface and propagate by dissolving the sphere structure (see supplementary material, Figure S1B). The same markers where then investigated in these cells after attachment and the expression pattern observed was comparable to the parental mono‐adherent cells (Figure 1E and see supplementary material, Figure S1B). Similarly, 769P, A498, and ACHN showed revertible EMT phenotype (data not shown).

Several recent studies have shown that hypoxic conditions enhanced stemness features 28, 29. Therefore, sphere formation capability was investigated under hypoxia (48 h, 0.2% O_2_, 5% CO_2_). An increased production of spheres was observed in parental cells upon hypoxic incubation, whereas sphere‐derived cells did not further enhance their sphere formation, potentially due to the constitutive expression of HIFs under normoxia (*p* < 0.05; see supplementary material, Figure S1C). Sphere‐derived cells showed increased levels of HIF1α and its downstream target CAIX compared to the corresponding parental cells. A498 and 769P did not show HIF1α expression due to homozygous deletion of HIF1α 30, instead they did express high levels of HIF2α (see supplementary material, Figure S1D). Of note, Caki‐1 and ACHN have a wild type (wt) *VHL*, whereas A498 and 769P carry a mutation (mut) in the *VHL* gene. These data not only show the positive effect of hypoxia in enhancing stem cell features but more importantly that both culture types, VHL wt and VHL mut, have overlapping stem cell properties, indicating that we found a general feature of ccRCC.

### Identification of potential novel cancer stem cell markers

**Figure 2:**
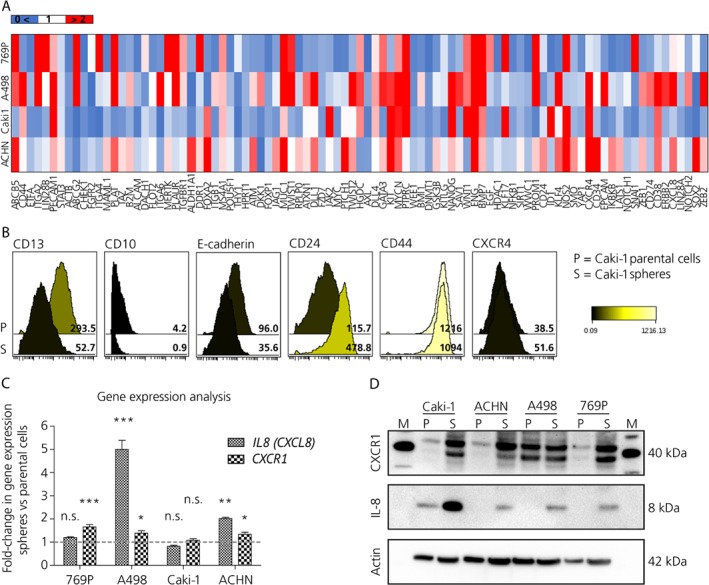
Renal cancer stem cells are characterized by IL‐8 and CXCR1 expression. (A) Heatmap depicting differential gene expression in the spheres compared to the corresponding parental cells using the Qiagen human CSC gene expression array. (B) CyTOF analysis of cellular expression of CD13, CD10, E‐cadherin, CD24, CD44, and CXCR4 in Caki‐1 spheres and parental cells. (C) Differential gene expression analyzed by RT‐qPCR for *IL8* and *CXCR1* in spheres compared to parental cells for 769P, A498, Caki‐1, and ACHN (one‐way ANOVA, *n* = 3). (D) Western blot for IL‐8 and CXCR1 in spheres and parental cells derived from ccRCC cell lines.

To identify potential novel CSC markers, a human CSC gene expression array analysis (RT^2^ Profiler PCR Array; Qiagen, Hilden, Germany), which profiles 84 genes linked to stemness, was performed on the spheres derived from 769P, A498, Caki‐1, and ACHN cells compared to the parental cells (Figure 2A). Differentially expressed genes are noted in Table 2. Changes in the gene expression profile such as upregulation of EMT and stemness markers and genes involved in developmental pathways (e.g. *NANOG*, *WNT*, and *MYCN*) were observed in sphere‐forming cells versus corresponding adherent parental cells. Moreover, several potential CSC markers including *CD105*, *CD133*, *CXCR4*, *ABCB5*, and *IL8*, were found to be highly expressed by the sphere population (above the threshold >1.2‐fold‐change).

**Table 2:** Differentially expressed genes in a gene expression array of renal cancer spheres and parental cells

CSC markers	Others	Stemness/developmental pathways	Marker of interest
CD105	CD38	ZEB1/2	IL‐8 (CXCL8)
CD133	BMP7	NANOG	
CXCR4	MUC1	WNT1	
ABCB5		MYCN	
		TWIST1/2	

We took advantage of cyTOF mass cytometry to simultaneously analyze the expression of different markers on the cell surface of adherent and sphere cell populations in the two metastatic RCC cell lines. Spheres showed an increased expression of EMT‐ and putative stem cell‐markers (CD24, CD44, and CXCR4) as well as a decreased expression of markers of cellular differentiation (CD13, CD10, and E‐cadherin), validating the mRNA analysis. The effect for Caki‐1 is illustrated in Figure 2B.

Among all of the candidates, we focused our attention on IL‐8 and CXCR1 since their role in ccRCC as potential CSC markers is currently unknown.

### IL‐8/CXCR1 axis is associated with cancer stem cell properties in ccRCC

In order to confirm the results obtained by the gene expression array, and to further dissect the role of the IL‐8/CXCR1 axis in ccRCC, RT‐qPCR for *CXCL8* and *CXCR1* was performed. Enhanced expression of *CXCL‐8* and *CXCR1* was observed in the sphere‐derived cells compared to the parental cells in all the cell lines analyzed except for Caki‐1 cells (Figure 2C). Similar results were obtained by western blot and immunohistochemical analysis except for CXCR1 in A498 cells (Figure 2D and see supplementary material, Figure S2A). Interestingly, Caki‐1 cells showed increased levels of IL‐8 and CXCR1 proteins which was not observed using RT‐qPCR (Figure 2D and see supplementary material, Figure S2A, B). However, Caki‐1 cells had high basal *CXCL8* expression levels, making any difference hard to detect. ELISA analysis of cell culture supernatants showed no difference in IL‐8 secretion for the spheres compared to parental cells in A498 cells (fold‐change: 1.02; n.s.). Whereas a positive but statistically not significant trend in IL‐8 secretion was observed in 769P (fold‐change: 1.3; n.s.) and, in particular, in the metastatic RCC cell lines Caki‐1 and ACHN (fold‐change: 4.7 and 1.45, respectively; n.s.; Figure S2C). These results were in line with the cytokine profile of cell culture supernatants derived from Caki‐1 and 769P (see supplementary material, Figure S2D, E and Table S2).

To further investigate the association of CXCR1 with CSC‐like properties in ccRCC cells, we established a flow cytometry assay to determine the expression levels of CXCR1 on Hoechst SP cells. Two lung cancer cell lines (H460 and A549) were first exploited to determine the gating strategy for CXCR1. The H460 cell line express CXCR1 at high levels 31, and served as positive control while A549 cells showed low CXCR1 levels (see supplementary material, Figure S2F).

The Hoechst SP analysis is one of the several strategies used to identify stem cell populations, and SP was identified as the portion of events that disappeared upon treatment with 100 μm verapamil which blocks the efflux of Hoechst in the CSCs (see supplementary material, Figure S2G). All parental ccRCC cell lines exhibited the presence of a Hoechst SP. The size of the SP was higher in the spheres than in parental cells in all four ccRCC cell lines tested (see supplementary material, Table S3). In most cell lines the abundance of cells in the total population that expressed CXCR1 was similar to or greater than the abundance of SP cells and importantly, between 23.1 ± 1.0% and 93.7 ± 8.9% of SP cells were positive for CXCR1, consistent with the notion that CXCR1 is a marker of CSCs (see supplementary material, Table S3). However, it was also apparent that some non‐SP cells can also express CXCR1 (see supplementary material, Table S3).

As previously reported in the literature, IL‐8 interacts also with CXCR2 32, and therefore we investigated whether CXCR2 expression levels may also be specifically associated with SP cells (see supplementary material, Figure S2H). In spheres from 769P, Caki‐1 and ACHN cells, CXCR2 was expressed by over 60% of the total population of cells (see supplementary material, Table S3). Therefore, the expression of CXCR2 does not correlate with the SP and we chose to pursue further functional analyses for CXCR1.

### IL‐8/CXCR1 axis is essential for sphere formation

To functionally analyze the role of IL‐8/CXCR1 signaling in CSC function we inhibited CXCR1 using a neutralizing anti‐CXCR1 antibody (20 μg/ml), or the small molecule repertaxin (100 nm). Human recombinant IL‐8 (1 μg/ml) was employed to enhance IL‐8 stimulation. Mildly increased cell proliferation of adherent cells was observed after 72 h treatment using human recombinant IL‐8 in all the ccRCC cell lines except for A498 (see supplementary material, Figure S3A–D). Conversely, addition of anti‐CXCR1 antibody reduced cellular proliferation of all cell lines except for Caki‐1 (see supplementary material, Figure S3A–D). Repertaxin treatment did not show any effect on cell proliferation (see supplementary material, Figure S3A–D). Invasion and migration properties were investigated using the real time cell analyzer XCELLigence (see supplementary material, Supplementary materials and methods). This technique adapts the Boyden chamber principle and combines it with impedance measurements. IL‐8 treatment was found to enhance cell invasion in 769P, Caki‐1, and ACHN cells (see supplementary material, Figure S3E,F), and cell migration in Caki‐1 and ACHN cells. On the contrary, CXCR1 blockade achieved by anti‐CXCR1 antibody or repertaxin showed opposite or no effect, whereas the combination of treatments showed intermediate increase of invasive and migratory properties indicating that IL‐8 may also act through other signaling pathways (see supplementary material, Figure S3E,F).

**Figure 3:**
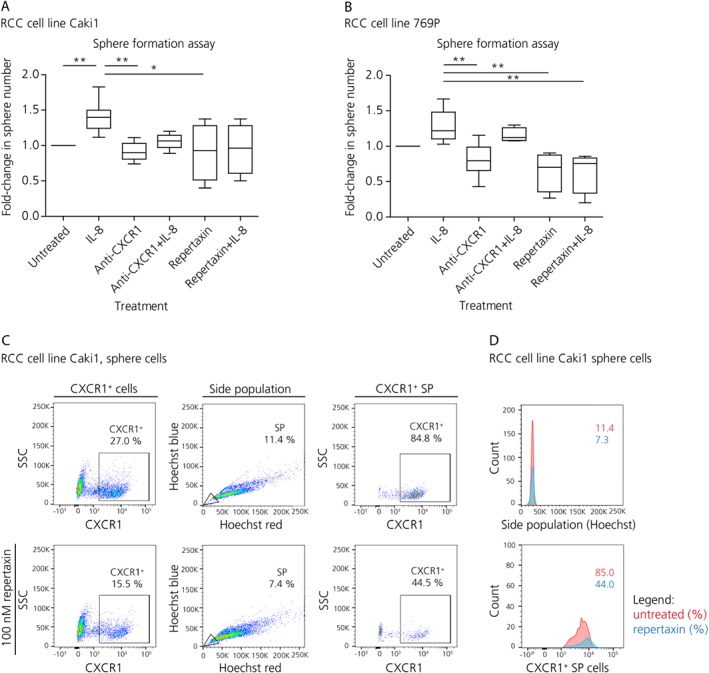
IL‐8/CXCR1 contributes to stemness *in vitro*. (A) Effect on sphere formation of single agent or combination treatment with human recombinant IL‐8, anti‐CXCR1 antibody and/or repertaxin for Caki‐1 (Kruskal–Wallis test, *n* = 5). (B) Effect on sphere formation of single agent or combination treatment with human recombinant IL‐8, anti‐CXCR1 antibody and repertaxin for 769P cells (Kruskal–Wallis test, *n* = 5). (C) Flow cytometry analysis of the SP and the CXCR1^+^ cell compartment upon repertaxin treatment in the spheres derived from Caki‐1. (D) Histograms showing decreased SP and CXCR1^+^ cells upon repertaxin treatment. The yellow area indicates number of events with verapamil treatment. The red area shows CSC and CXCR1^+^ populations in the untreated sample. The blue area displays the remaining number of events after repertaxin treatment.

To test the effect of IL‐8/CXCR1 on sphere formation, cells were incubated for 72 h in the sphere formation assay with anti‐CXCR1 antibody, repertaxin or IL‐8 treatments. IL‐8 treatment increased sphere content by around 1.5‐fold (A‐498 *p* < 0.05; 769P *p* = n.s.; Caki‐1 *p* < 0.02; ACHN *p* < 0.05). The effect on Caki‐1 and 769P is illustrated in Figure 3A,B. A negative but statistically not significant trend in sphere formation compared to untreated control was observed upon anti‐CXCR1 antibody treatment (*p* = n.s.); whereas its combination with IL‐8 showed no effect (*p* = n.s.). Similarly, repertaxin alone or in combination with IL‐8 did not show any major effect on sphere formation except for 769P where it significantly decreased sphere formation when compared to IL‐8 treatment (*p* < 0.02; Figure 3A,B).

Flow cytometry analysis revealed that 72 h treatment with repertaxin significantly decreased SP and CXCR1^+^cells in the spheres as well as in the parental samples (see supplementary material, Figure S4A–D and Table S4). For instance, in Caki‐1 spheres, the CXCR1^+^SP decreased by about 50% upon repertaxin treatment going from 84.8 to 44.5% CXCR1^+^cells (Figure 3C,D).

Taken together these results indicate to us that IL‐8 stimulates cell proliferation and invasion as well as CSC formation and self‐renewal. In contrast, CXCR1 blockade decreases cell proliferation, cell invasion and partially affected CSC properties. This indicates that IL‐8 stimulatory effect on sphere formation is at least partly dependent on CXCR1.

### IL‐8/CXCR1 axis plays an important role in tumor development and metastasis formation *in vivo*

**Figure 4:**
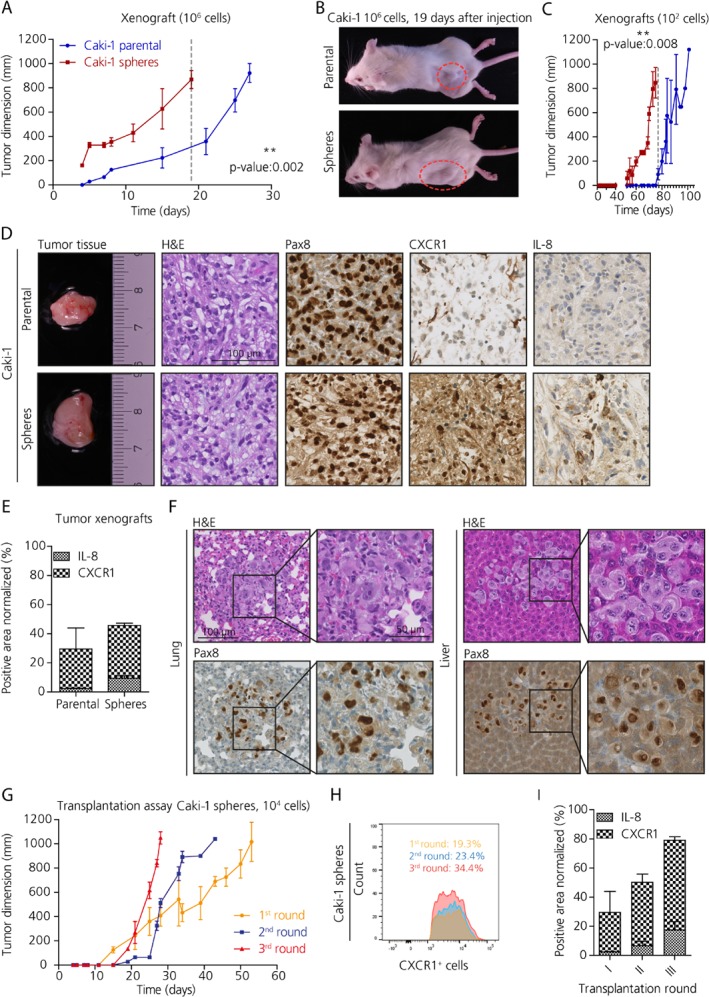
IL‐8/CXCR1 signaling contributes to tumor development and metastasis formation *in vivo*. (A) Tumor growth evaluation in NSG xenografts subcutaneously injected with 10^6^ cells derived from Caki‐1 spheres (in red) and parental cells (in blue) (unpaired *t*‐test, *n* = 3). (B) Representative pictures of immune compromised mice at 19 days post‐injection with either parental or sphere cells. (C) Tumor growth evaluation in NSG xenografts subcutaneously injected with 10^2^ cells derived from Caki‐1 spheres (in red) and parental cells (in blue) (unpaired *t*‐test, *n* = 3). (D) Sections of xenografted tumors derived from Caki‐1 showing the histological subtype by H&E, and the kidney nature PAX8 positivity, and differential CXCR1 and IL‐8 expression in the tumors derived from spheres compared to those derived from parental cells. Scale bar: 100 μm. (E) Quantification of immunohistochemical stains for IL‐8 and CXCR1 in tumor xenografts derived from Caki‐1. (F) Micrometastases in lungs and liver of xenografts derived from the injection with 10^4^ Caki‐1 sphere cells. Scale bar: 100 μm, 50 μm. (G) Transplantation assay (*n* = 3). (H) Analysis using FACS of the CXCR1^+^ population in xenografted tumors following retransplantation of xenografted tumors. (I) Quantification of immunohistochemical staining for CXCR1 in tumor xenografts derived from Caki‐1 upon retransplantation of xenografted tumors (*n* = 2).

Sphere‐derived Caki‐1 cells as well as parental Caki‐1 cells gave rise to tumors when injected into NSG (NOD.Cg‐Prkdcscid Il2rgtm1Wjl/SzJ) mice at three different cell numbers (10^6^, 10^4^, 10^2^). However, in each case tumor formation and growth was enhanced when tumor cells derived from the spheres were injected compared to parental cells. For instance, Caki‐1 spheres injected at 10^6^ cells reached the tumor size of 1 cm^3^ after only 19 days, whereas mice injected with parental cells harbored tumors less than half of the diameter (358.1 ± 187.4 mm) after the same time period (*p* = 0.002; Figure 4A,B).

Mice injected with 10^2^ parental cells did eventually start to develop tumors at the site of injection after 70 days post‐injection, a time point at which tumors derived from spheres were already harvested because they reached the maximum permitted tumor bearing size (*p* = 0.008; Figure 4C). The Caki‐1 cell line is derived from a metastatic tumor; therefore, these cells are more aggressive and prone to tumor formation. In fact, metastasis‐derived cultures contained more stem cells than primary tumor‐derived cell lines. Further support for the notion that growth as spheres enriches for CSC activity is that injection of 10^2^, 10^4^ or 10^6^ sphere‐derived cells from 769P developed tumors when injected into NSG mice, but no tumors formed for up to 130 days post‐injection when parental cells were injected (see supplementary material, Figure S5A,B).

Histologically, xenografted tumors resembled the ccRCC subtype and were positive for PAX8, a transcription factor expressed in epithelial cells of the adult kidney and in about 90% of renal cell neoplasms (Figure 4D; see supplementary material, Figure S5C). Interestingly, tumors derived from Caki‐1 spheres showed higher CXCR1 and IL‐8 expression by IHC compared to tumors derived from the corresponding parental cells (Figure 4D,E and see supplementary material, Figure S5C). IL‐8/CXCR1 cells were confined within certain areas of the tumor, but did not cluster together.

Mice injected with Caki‐1 or 769P sphere‐derived cells developed micrometastases in the lungs and liver, whereas mice injected with parental cells did not show micrometastasis (Figure 4F and see supplementary material, Figure S5D,E). Interestingly, macrometastases were identified when mice received 10^4^ but not 10^6^ sphere‐derived cells This is most likely due to the much shorter time required for primary tumors to reach 1 cm^3^ in mice that received 10^6^ cells, at which point the mice were sacrificed (see supplementary material, Figure S5F; Table 3). In contrast, mice injected with 10^2^ cells did not show any macrometastases, probably due to the low number of cells injected.

**Table 3:** Evaluation of distant metastasis in ccRCC xenografts

Number of cells injected	Time for tumor of 1 cm^3^	Micrometastasis	Macrometastasis	Number of cells injected	Time for tumor of 1 cm^3^	Micrometastasis	Macrometastasis
**Caki‐1 parental**				**Caki‐1 spheres**			
10^6^	27 days	−	−	10^6^	19 days	+	−
10^4^	63 days	+	−	10^4^	53 days	+	+
10^2^	101 days	−	−	10^2^	83 days	+	−
**769P parental**				**769P spheres**			
10^6^	−	n.a.	n.a.	10^6^	28 days	+	−
10^4^	−	n.a.	n.a.	10^4^	46 days	+	+
10^2^	−	n.a.	n.a.	10^2^	69 days	+	−

n.a., not assessed because of the absence of primary tumor.

Xenografted primary tumors were isolated from mice as soon as they had grown to 1 cm^3^ and were dissociated; 10^4^ cells were then re‐injected into new NSG mice. Xenografted primary tumors were re‐transplanted for a further two generations. Strikingly, enhanced tumor growth was observed upon each re‐transplantation (Figure 4G). Each time the primary xenograft was harvested and dissociated, part of the single cell suspension generated was used to analyze the CXCR1^+^ population by flow cytometry. Interestingly, CXCR1^+^ cells also increased in abundance upon each re‐transplantation. For instance, CXCR1^+^ cells formed 19.3% of the dissociated tumor cells derived from Caki‐1 sphere xenografts in first round of transplants, and that proportion increased to 23.4% in the second round, and finally reached 34.4% in the third round of transplantation (Figure 4H,I). At the same time, another portion of the dissociated tumor tissue was plated into the sphere formation assay. As expected, tumor cells were capable of reforming spheres in culture, indicating that they retained stem cell features (data not shown). Additionally, human IL‐8 levels in the sera of xenografts was measured by ELISA. Mice harboring no tumors (769P parental) showed IL‐8 levels comparable to the control mice, whereas sphere‐derived xenografts (Caki‐1) showed higher IL‐8 levels in the serum compared to xenografts of parental cells (see supplementary material, Figure S5G, *n* = 3).

### IL‐8/CXCR1 expression correlates with poor prognosis in renal cancer patients

**Figure 5:**
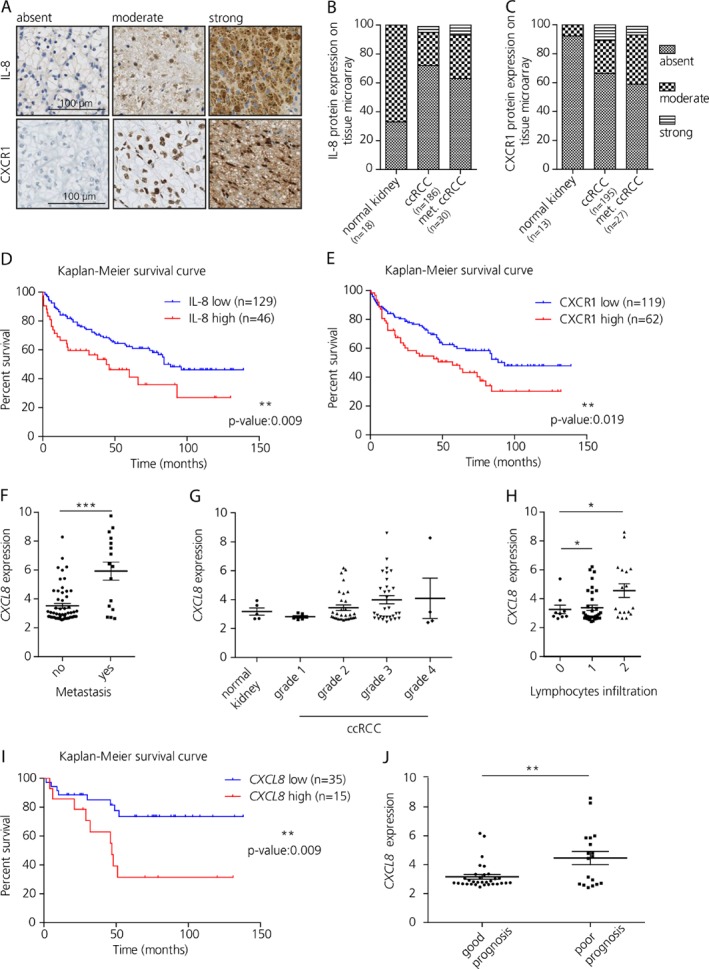
IL‐8 and CXCR1 expression correlates with clinico‐pathological features in ccRCC patients. (A) IL‐8 and CXCR1 expression on tissue microarrays categorized in absent, moderate and strong according to the staining intensity. Scale bar: 100 μm. (B) Quantification of staining for IL‐8 in tissue microarrays. (C) Quantification of staining for CXCR1 in tissue microarrays. (D) Kaplan–Meier survival curve for IL‐8 [low: absent; high: moderate and strong); log‐rank (Mantel–Cox) test]. (E) Kaplan–Meier survival curve for CXCR1 [low: absent; high: moderate and strong; log‐rank (Mantel–Cox) test]. (F) Analysis of *CXCL8* RNA expression on metastatic ccRCC compared to nonmetastatic ccRCC (unpaired *t*‐test; *n* = 57 and 32, respectively). (G) Relative mRNA expression of *CXCL8* according to ccRCC grade. (H) Analysis of *CXCL8* mRNA expression according to tumor lymphocytes infiltration (one way ANOVA). (I) Kaplan–Meier survival curve calculated according to the median *CXCL8* mRNA expression [log‐rank (Mantel–Cox) test]. (J) RNA expression for *CXCL8* according to 5 year overall survival (*t‐*test).

In order to investigate the translational relevance of the IL‐8/CXCR1 axis to the clinic, IL‐8 and CXCR1 protein expression was evaluated in 255 ccRCC patients using tissue microarrays (Table S5 and see supplemental material, Supplementary material and methods). Staining intensities were classified as absent, moderate and strong (Figure 5A–C). We found a striking negative correlation between IL‐8 and CXCR1 with overall survival (OS) of ccRCC patients (*P* values of 0.009 and 0.019, respectively; Figure 5D,E). In particular, the median OS for ccRCC patients with high IL‐8 levels was 48 months, whereas patients with low IL‐8 levels showed a median OS of 84 months (Figure 5D). High CXCR1 levels showed a median OS of 57 months, whereas low CXCR1 showed a median OS of 93 months (Figure 5E).

Additionally, the level of *CXCL8* mRNA was investigated in 85 patients affected by ccRCC that had been reported previously 33 (Table S5 and see supplemental material, Supplementary material and methods) The RNA expression was compared to matched normal kidney tissue (*n* = 5). Significantly higher *CXCL8* expression was found in metastatic ccRCC (*n* = 20; *p* = 0.0001; Figure 5F) compared to normal tissue, and a positive trend was observed in relation to tumor grade and advanced stage in ccRCC patients (Figure 5G). In addition, *CXCL8* was found positively correlated with strong tumor lymphocytes infiltration (*p* = 0.0125; Figure 5H), and decreased overall survival (*p* = 0.009; Figure 5I,J).

Taken together, the IL‐8/CXCR1 axis represents a marker for poor prognosis in ccRCC.

## Discussion

In this study, we isolated a small population of cells from ccRCC cell lines expressing high levels of the chemokine IL‐8 and its receptor CXCR1. Interestingly, we found that IL‐8/CXCR1 expression was associated with CSC‐like properties *in vitro* and *in vivo*. Further, *CXCL8* expression correlated with intratumoral lymphocytic infiltration and decreased overall survival in ccRCC patients.

In the present work, functional assays and *in vivo* models were used to identify and characterize renal CSCs. Cancer cells isolated by the sphere formation assay displayed self‐renewal properties, upregulation of EMT markers (vimentin, Snail/Slug and N‐cadherin), stemness and developmental genes (*CD105*, *CD133*, *CXCR4*, *NANOG*, *MYCN*, and *WNT*). In addition, spheres expressed low levels of E‐cadherin and markers of renal differentiation such as CD10 and CD13.

Higher levels of the chemokine IL‐8 and its receptor CXCR1 but not CXCR2 were found at the RNA level, and at the protein level by Western blot, IHC and flow cytometry. Additionally, a high concentration of IL‐8 was found in cell supernatants particularly of the spheres derived from metastatic RCC cell lines. IL‐8 and CXCR1/2 have recently been demonstrated to be associated with CSC populations in many tumor types such as breast, prostate, colon and pancreatic cancers 34, 35, 36. However, CXCR1 and CXCR2 were found expressed independently or together in different tumor types, indicating they may have different influence on CSC activity 34, 37, 38.

In our study, significantly higher *CXCL8* levels were found in metastatic ccRCCs compared to normal tissue, and a positive trend was observed in relation to tumor grade and stage in ccRCC patients. Moreover, IL‐8/CXCR1 expression correlated decreased overall survival. Similarly, increased IL‐8 and CXCR1 expression was shown to be an independent adverse prognostic factor in patients with ccRCC after nephrectomy 39, 40.

We revealed that ccRCC cultures treated with CXCR1 blocking agents (anti‐CXCR1 antibody and repertaxin) exhibited reduced proliferation, migration and invasion, sphere formation and self‐renewal properties, whereas IL‐8 stimulation enhanced CSC properties. This is in line with previous studies in HCC, breast and pancreatic cancers where anti‐IL‐8/CXCR1 impaired CSC features 34, 35, 41. Moreover, flow cytometry analysis revealed that repertaxin treatment significantly decreased the SP and CXCR1^+^ cells in the spheres as well in the parental samples. When injected subcutaneously into NSG mice, sphere‐derived cells gave rise to tumors and metastasis in 20 days. Whereas, parental cells derived exclusively from the metastatic ccRCC culture Caki‐1 were only able to form tumors after over 100 days. Therefore, sphere‐derived cells correlate with *in vivo* metastasis and that is consistent with the fact that cell cultures derived from metastasis showed higher sphere forming capacity and stemness features compared to primary tumor‐derived cells. Mouse models of cancer play a vital role in understanding tumor biology 42. To date, *in vivo* models of metastatic ccRCCs are either lacking or inadequate; here we generated a valuable resource for scientists interested in dissecting the process of metastasis in RCC.

Interestingly, high IL‐8 and CXCR1 levels were found in the xenograft tumor tissue derived from spheres as well as elevated IL‐8 concentrations in serum of these xenografts. This is in line with our finding that CXCR1 expression as well as tumor development was enhanced upon tumor transplantation. These results suggest that transplantation fosters the expansion of CSCs, thus, CXCR1^+^ populations may play a key role in tumor progression.

Taken together, our results suggest that the IL‐8/CXCR1 axis is associated with CSC‐like properties in renal cancer with implications for renal cancer treatment. Targeting IL‐8 in combination with conventional chemotherapy agents and/or immunotherapy might prove to be the next step towards overcoming tumor recurrence in ccRCC. Nevertheless, further investigations corroborating the therapeutic applicability of IL‐8/CXCR1 are required.

## Author contributions statement

CC together with MR and HM conceived the original idea. CC developed the necessary methodology, generated tumor spheroids, designed and performed *in vitro* and *in vivo* experiments. CC was responsible for all data acquisition, analysis and interpretation as well as manuscript writing. MEH performed *in vivo* assays, reviewed the manuscript and provided critical suggestions during the project execution. SE and BB carried out mass spectrometry analysis of the samples. BB, ZL, PS, IF, and AW reviewed the manuscript and provided critical support during experimental design. HM reviewed all renal tumor specimens and neoplasms. Finally, HM and MR provided conceptual support, assisted in the manuscript writing, and supervised the study. All authors discussed the results and commented on the manuscript.

**SUPPLEMENTARY MATERIAL ONLINE**

**Supplementary materials and methods**

**Supplementary figure legends**

**Figure S1.** Sphere‐propagating cells display stem‐like properties

**Figure S2.** Side population cells are characterized by CXCR1 expression

**Figure S3.** IL‐8/CXCR1 signaling affects cell proliferation, migration and invasion in ccRCC cell lines

**Figure S4.** Repertaxin treatment reduced SP and CXCR1+ cells

**Figure S5.** Tumor xenografts derived from the 769P cell line

**Table S1.** Detailed antibody information

**Table S2.** Human cytokine array panel

**Table S3.** Flow cytometry data for SP and CXCR1/2+ cell populations in parental and sphere cells in ccRCC cell lines

**Table S4.** Flow cytometry data for SP and CXCR1+ cells in parental and spheres before and after repertaxin treatment

**Table S5.** Patients' details for TMA and RNA analyses

## Supporting information


**Supplementary material and methods**

**Supplementary figure legends**

**Figure S1.** Sphere‐propagating cells display stem‐like properties

**Figure S2.** Side population cells are characterized by CXCR1 expression

**Figure S3.** IL‐8/CXCR1 signaling affects cell proliferation, migration and invasion of ccRCC cell lines

**Figure S4.** Repertaxin treatment reduced SP and CXCR1^+^ cells

**Figure S5.** Tumor xenografts derived from the 769P cell line

**Table S1.** Detailed antibody information
**Table S2.** Human cytokine array panel
**Table S3.** Flow cytometry data for SP and CXCR1/2+ cell populations in parental and sphere cells in ccRCC cell lines
**Table S4.** Flow cytometry data for SP and CXCR1+ cells in parental and spheres before and after repertaxin treatment

**Table S5.** Patients' details for TMA and RNA analyses
