# Supplementary material for: IL‐8 and CXCR1 expression is associated with cancer stem cell‐like properties of clear cell renal cancer
Source: J Pathol. 2019 Apr 11;248(3):377–89. doi: 10.1002/path.5267 (PMC6618115; doi:10.1002/path.5267)
Supplement: Supplementary file 1 — Supplementary material and methods [file PATH-248-377-s006.docx]

**IL-8 and CXCR1 expression is associated with cancer stem cell-like properties of clear cell renal cancer**

Corrò C *et al*. *J Pathol* DOI: 10.1002/path.5267

**Supplementary material and methods**

Reference numbers refer to the main text list

Animal Housing

Animals were maintained under specific pathogen free (spf) conditions with a light/dark cycle of a 12 h/12 h cycle with artificial light of approximately 40 lux in the cage. The mean room temperature was 21 ± 1 °C, with a relative humidity of 50 ± 5 % and 15 complete changes of filtered air per hour (HEPA H14 filter); the air pressure was controlled at 50 Pa.

Patient cohort information

In our study, we profiled protein expression using tissue microarrays containing tumor tissue derived from 255 ccRCC specimens and 29 normal kidney tissues. The samples were retrieved from the archives of the Department of Pathology and Molecular Pathology; University Hospital Zurich (Zurich, Switzerland) between the years 1993 to 2003. The age of patients ranged between 31 and 88 years old (mean age: 64 years old) and the female:male ratio was 1:3.

RNA expression was investigated in 85 patients affected by ccRCC and compared to 5 matched normal specimens. The samples were retrieved from the archives of the Department of Pathology and Molecular Pathology; University Hospital Zurich (Zurich, Switzerland) between the years 1992 to 2007. The age of patients ranged between 22 and 86 years (mean age: 62 years) and the female:male ratio was 1:3.

Cell lines

The ccRCC cell lines 769P, A498, Caki-1 and ACHN together with the lung cancer cell lines A549 and H460, were purchased from ATCC (Manassas, VA, USA). These cell lines were additionally authenticated by STR-based DNA-profiling by IdentiCell (Department of Molecular Medicine, Aarhus University, Denmark). Cells were cultured using Dulbecco’s Modified Eagle Medium (DMEM, Thermo Scientific Inc., Waltham, MA, USA) or RMPI medium containing 10 % FCS and 2 mM glutamine (Thermo Scientific). Cells were split when reaching 80 % confluence.

Cell invasion and migration

Migratory/invasive potential of ccRCC cell lines was measured in real-time using the xCELLigence RTCA DP System (ACEA Biosciences, San Diego, CA, USA). This technique adapts the Boyden chamber principle and combines it with impedance measurements. Cells seeded in the upper chamber of a microplate containing “low chemoattractant (1 % FCS)” can migrate through the microporous membrane into the lower chamber being the “high chemoattractant (10 % FCS)” compartment. Migrated cells adhere to the gold micro-electrode sensor located at the lower side of the membrane and lead to an increase in impedance, which is measured by the RTCA DP instrument. Optimal cell seeding densities were determined in pre-experiments and 20,000 cells were found to be optimal. Impedance measurements were performed for 72–96 h. For invasion assays, the membrane was coated with Matrigel Basement Membrane Matrix (BD Biosciences, San Jose, CA, USA; 400 µg/ml protein in 1 % FCS-containing medium).

Hypoxia

For hypoxic treatment, cells were cultured for 48 h at 0.2 % oxygen in an INVIVO2 hypoxia workstation 300 (Ruskin Technology, Bridgend, UK).

Tumorsphere culture

Generation of tumorspheres was performed by resuspending 5x10^4^ cells into a special medium containing DMEM/F12 Thermo Scientific Inc.), 2 % B27 supplement (Thermo Scientific Inc.), 20 ng/mL Human Recombinant EGF (Thermo Scientific Inc.), 10 ng/mL Recombinant Human FGF-basic (Thermo Scientific Inc.), 5 µg/ml Insulin (Sigma-Aldrich, St. Louis, MO, USA) and 10 % FCS. Cells were then plated into ultra-low attachment 10 cm^2^ dishes (Corning, Corning, New York, USA) and the medium was refreshed every 4 days. Tumorspheres at approximately 300 μm in size were collected by centrifugation (500 rpm, 5 min) into 50 ml Falcon tubes. The supernant was carefully removed and spheres were washed using PBS. Spheres were passaged by incubation with 1 ml of TrypLE Express (Thermo Scientific Inc.) for 5 min in an incubator at 37 °C. Spheres were further dissociated by slowly pipetting up and down the cell suspension. The trypsin was then inactivated by adding at least three volumes of DMEM containing 10 % FCS and the cells were pelleted by centrifugation at 500 rpm for 5 min. Single cell suspension was re-plated according to the procedure described above.

Limiting dilution assay

Sphere formation capability was evaluated by limiting dilution assay (LDA). Adherent tumor cells and sphere-derived cells were plated in triplicates into 96-well plates at 0.5 cell/well density in the tumor sphere medium. Only wells which contained 1 cell/well were marked and observed every day and 10 µl of fresh medium was added every 2 days. After a week, tumor sphere formation was evaluated in the marked wells using the formula: (n of positive wells/n of total marked wells) x 100.

Clonogenic assay

Cells were harvested, counted, and diluted in sterile tubes, in order to plate 200 cells/well into 6-well plates or 2,000 cells into 10 cm^2^ dishes. Appropriate medium was used to grow cells over two-week time period. Fresh medium was added every 3 days to avoid cell death due to senescence. In the treatment experiment, cells were pretreated for 72 h before seeding them into the clonogenic assay. After two weeks, medium was removed and cells were rinsed carefully with PBS. Methanol was used to fix the cells for 10 min following staining with 0.5 % crystal violet for 15 minutes. The excess dye was removed by rinsing each well with tap water. Dishes and plates were left to dry in normal air at RT. Colonies containing up to 50 cells were counted under the microscope.

RT-qPCR

Total RNA was purified using the Maxwell 16 RNA Purification Kit (Promega, Madison, WI, USA) and quantified using the Qubit Protein Assay Kit (Thermo Scientific Inc.). Total RNA (400 ng) was reverse‐transcribed using the RT2 First Strand Kit (Qiagen, Hilden, Germany). A human CSC gene expression array (RT^2^ Profiler PCR Array; Qiagen, Hilden, Germany), which profiles 84 genes linked to CSCs, was employed. In addition, the expression of *CXCL8* mRNA was verified separately using the RT^2^ qPCR Primer Assay for *IL8*, NM_000584. The QuantiTect Primer Assay was employed for *CXCR1*, NM_000634. Analysis was performed on qPCR machine (ViiA7 Real‐Time PCR System, Applied Biosystems, Thermo Scientific Inc.) in a 10 μl final volume.

Flow cytometry

10^6^ tumor cells were resuspended into 1 ml of DMEM containing 2 % FCS and 10 mM HEPES (Thermo Scientific Inc.) together with Hoechst 33342 (H3570, Thermo Fisher Scientific Inc.) at a final concentration of 5 µg/ml. Cells were then incubated at 37 °C in a water bath for 90 min. To confirm that the correct cells are identified as SP on the flow cytometer, an aliquot of cells was treated using 50 µM verapamil (V4629, Sigma-Aldrich) to block the efflux of Hoechst in the SP prior to incubation in the Hoechst staining solution. With verapamil treatment, SP disappears from the plot. After 90 min, cells were centrifuged at 500 rpm and resuspended in ice-cold HBSS containing 2 % FCS and 10 mM HEPES. Samples were further stained with either anti-CXCR1 (Alexa Fluor 700, FAB330N, R&D Systems, Minneapolis, MN, USA) or anti-CXCR2 (PerCP, FAB3331C, R&D Systems) antibody for 1 h at 4 °C and subsequently fixed in 1.6 % PFA (Thermo Scientific Inc.) for 10 min. For cell dead discrimination, 2 µg/ml propidium iodide (PI) was added immediately before flow cytometry. Hoechst dye was excited at 405 nm and the blue fluorescence signal was collected using a 450/40 nm band-pass filter and the red fluorescence with a 610/20 nm filter. Due to the high capability to efflux Hoechst dye, the SP can be defined as the population negative for Hoechst blue and Hoechst red. CXCR1 was detected by using a laser at 640 nm and a 730/45 nm emission filter, and for CXCR2, a laser at 488 nm with a 710/50 nm emission filter.

Mass cytometry

Formalin-fixed cell lines (Caki-1 and ACHN) and corresponding spheres were resuspended with Cell Staining Medium (CSM) with 0.3 % Saponin (CSM-S) and loaded into a 96-well plate. Samples were washed with PBS with 0.03 % Saponin (PBS-S) and barcoded with barcoding reagent (^104^Pd, ^106^Pd, ^108^Pd, ^110^Pd, ^113^In, ^115^In ) at a final concentration of 100 nM for 45 min at RT [43]. Samples were washed three times in CSM-S. Afterwards barcoded cells and spheres were combined and stained with a metal-conjugated antibody mix (supplementary material, Table S1) diluted in CSM-S for 1 h at 4 °C. Sample was washed three times with CSM-S and once with PBS-S. Samples were intercalated and fixed using MaxPar Intercalator 500 µM (Fluidigm, San Francisco, CA, USA) diluted 1:10,000 in 1.6 % PFA in PBS overnight at 4 °C. Samples were washed three times in PBS. Before analysis, the samples were washed once with purified water and filtered through a 35 µm cell strainer.

Samples were mixed 1:10 with EQTM Four Element Calibration Beads (Fluidigm) and then analyzed on a CyTOF2. Following the manufacturer’s standard operation procedure, samples were acquired at a rate of ~500 cells/s. After acquisition all generated fcs files were concatenated [43], then data were normalized and bead events removed [44], followed by a debarcoding step [45]. The data analysis was done using Cytobank (<http://www.cytobank.org/>). Therefore, cells were gated using ^191^Ir and ^193^Ir to remove doublets and debris.

ELISA

Cells were plated into the sphere formation assay for two weeks until spheres were formed. Afterwards, fresh media was added and 48 h later cell culture supernatants were collected. IL-8 concentration measured by sandwich ELISA (D8000C, R&D Systems, Minneapolis, MN, USA) according to the manufacturer's instructions. Sphere-derived cell supernatants were compared to the media collected from parental cells and to fresh medium. The IL-8 ELISA was also used for testing sera of xenografts harboring tumors and control mice (n=3).

Human cytokine array

Cells were plated into the sphere formation assay for two weeks until spheres were formed. Afterwards, fresh media was added and 48 h later cell culture supernatants were collected and analyzed for several human cytokines and chemokines using an antibody array (Proteome profiler ARY005B, R&D Systems) according to the manufacturer's instructions. Pixel density was analyzed using ImageJ.
