# Supplementary material for: IL‐8 and CXCR1 expression is associated with cancer stem cell‐like properties of clear cell renal cancer
Source: J Pathol. 2019 Apr 11;248(3):377–89. doi: 10.1002/path.5267 (PMC6618115; doi:10.1002/path.5267)
Supplement: Supplementary file 2 — Supplementary figure legends [file PATH-248-377-s007.docx]

**IL-8 and CXCR1 expression is associated with cancer stem cell-like properties of clear cell renal cancer**

Corrò C *et al*. *J Pathol* DOI: 10.1002/path.5267

**Supplementary figure legends**

**Figure S1.** Sphere-propagating cells display stem-like properties

(A) Representative pictures from the colony formation assay for Caki-1 spheres and parental cells. (B) Bright field images and immunohistochemical stains showing mesenchymal-epithelial transition (MET) upon attachment of the sphere into adherent conditions. Scale bar: 100 µm. (C) Sphere formation under hypoxia. (D) Western blot for HIF1α, HIF2α and CAIX in spheres (S) and parental cells (P).

**Figure S2.** Side population cells are characterized by CXCR1 expression

(A) Western blot quantification (n=3). (B) Immunohistochemistry for IL-8 and CXCR1. Scale bar: 100 µm. (C) Concentrations of IL-8 in supernatants of sphere and parental cell cultures determined by ELISA. (D) Dot blot cytokine microarray. (E) Human cytokine and chemokine profile of Caki-1 and 769P cell lines (spheres versus parental cells) (F) Analysis of CXCR1^+^ populations in two lung cancer cell line (H460 and A549) using FACS. (G) Analysis of SP cells expressing CXCR1 in Caki-1 spheres based on the exclusion of the DNA binding dye, Hoechst 33342, with and without verapamil using FACS. (H) Analysis of CXCR2^+^ cells in Caki-1 spheres using FACS.

**Figure S3.** IL-8/CXCR1 signaling affects cell proliferation, migration and invasion of ccRCC cell lines

(A) Analysis of cell proliferation of Caki-1 cells (two-way ANOVA, n=3). (B) Analysis of proliferation of 769P cells (two-way ANOVA, n=3). (C) Analysis of proliferation of ACHN cells (two-way ANOVA, n=3). (D) Analysis of proliferation of A498 cells (two-way ANOVA, n=3). (E) Analysis of invasion and migration of Caki-1 cells (two-way ANOVA, n=2). (F) Analysis of invasion and migration of 769P cells (two-way ANOVA, n=2).

**Figure S4.** Repertaxin treatment reduced SP and CXCR1^+^ cells.

(A) Histograms showing the SP and CXCR1^+^ cells upon repertaxin treatment for Caki-1 parental cells. The yellow area indicates the number of events with verapamil treatment. The red and blue areas represent the population before and after treatment, respectively. (B) Histograms showing the SP and CXCR1^+^ cells upon repertaxin treatment for 769P parental cells and spheres. The yellow area indicates the number of events with verapamil treatment. The red and blue areas represent the population before and after treatment, respectively. (C) Histograms showing the SP and CXCR1^+^ cells upon repertaxin treatment for A498 parental cells and spheres. The yellow area indicates the number of events with verapamil treatment. The red and blue areas represent the population before and after treatment, respectively. (D) Histograms showing SP and CXCR1^+^ cells upon repertaxin treatment for ACHN parental cells and spheres. The yellow area indicates the number of events with verapamil treatment. The red and blue areas represent the population before and after treatment, respectively.

**Figure S5.** Tumor xenografts derived from the 769P cell line.

(A) Evaluation of the tumor growth in NSG xenografts subcutaneously injected with 10^6^, 10^4^ and 10^2^ cells derived from 769P spheres (n=3). (B) Representative images of immune compromised mice injected with either Matrigel alone (control), or Matrigel containing parental or sphere cells. (C) Stained sections of xenograft tumor derived from 769P spheres showing the histological subtype by H&E, the kidney nature by PAX8 positivity, and CXCR1 and IL-8 expression. Scale bar: 100 µm. (D) H&E and PAX8 immunostained sections of lung and liver from control mice and mice injected with 10^6^ 769P parental cells. Scale bar: 100 µm. (E) Tumor cell extravasation in the lung tissue in a xenografted mouse derived from 769P spheres. Scale bar: 50 µm. (F) Micro-metastases in lung, liver, brain and lymph nodes of xenografts derived from the injection with 10^4^ 769P sphere cells. Scale bars: 100 µm and 50µm. (G) Concentrations of IL-8 in supernatants of xenografts derived from sphere and parental cells determined by ELISA and compared to control mice (n=3).
