# Supplementary figures and images for: IL‐8 and CXCR1 expression is associated with cancer stem cell‐like properties of clear cell renal cancer
Source: J Pathol. 2019 Apr 11;248(3):377–89. doi: 10.1002/path.5267 (PMC6618115; doi:10.1002/path.5267)

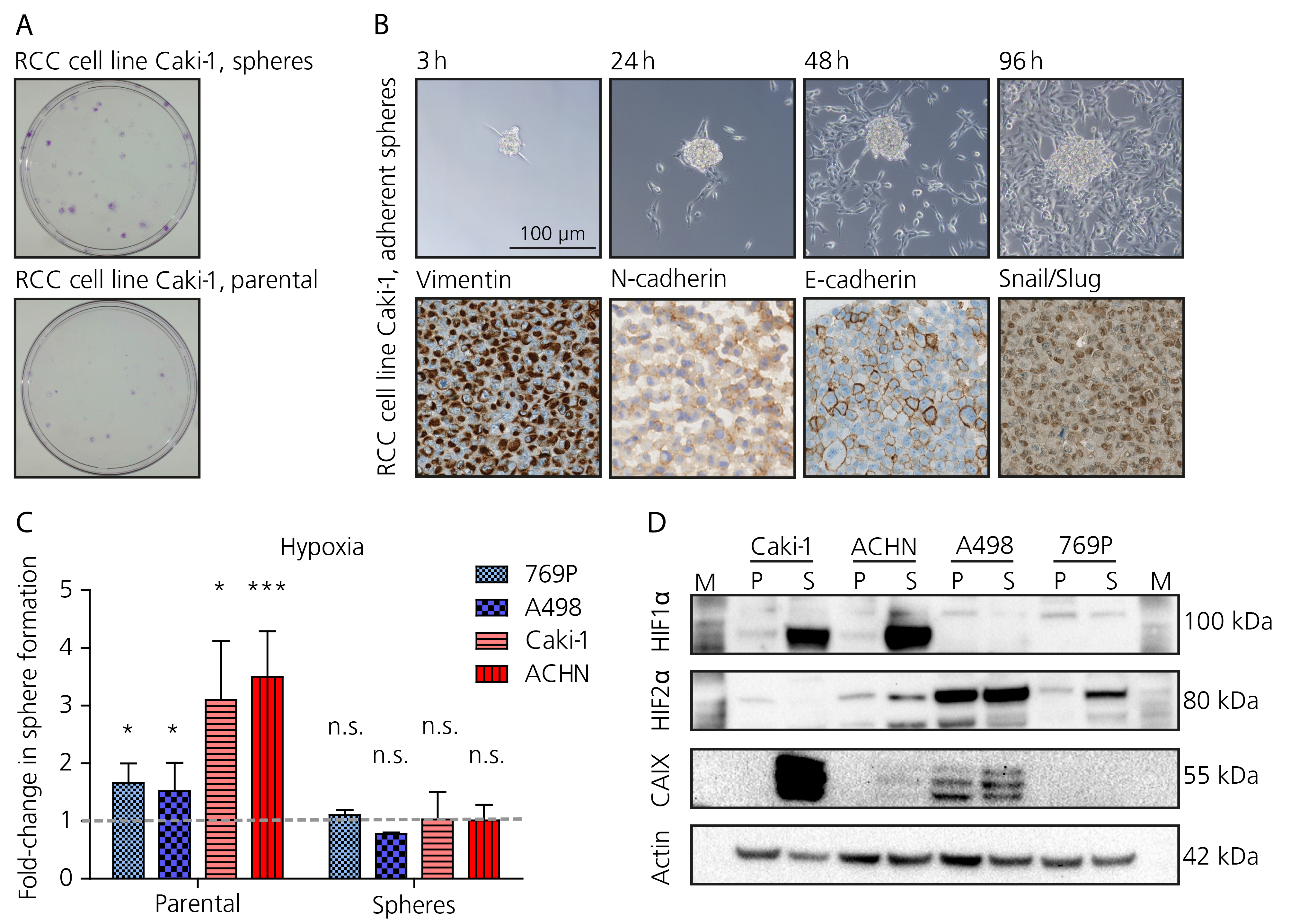

Supplement: Supplementary file 3 — Figure S1. Sphere‐propagating cells display stem‐like properties [file PATH-248-377-s001.tif]

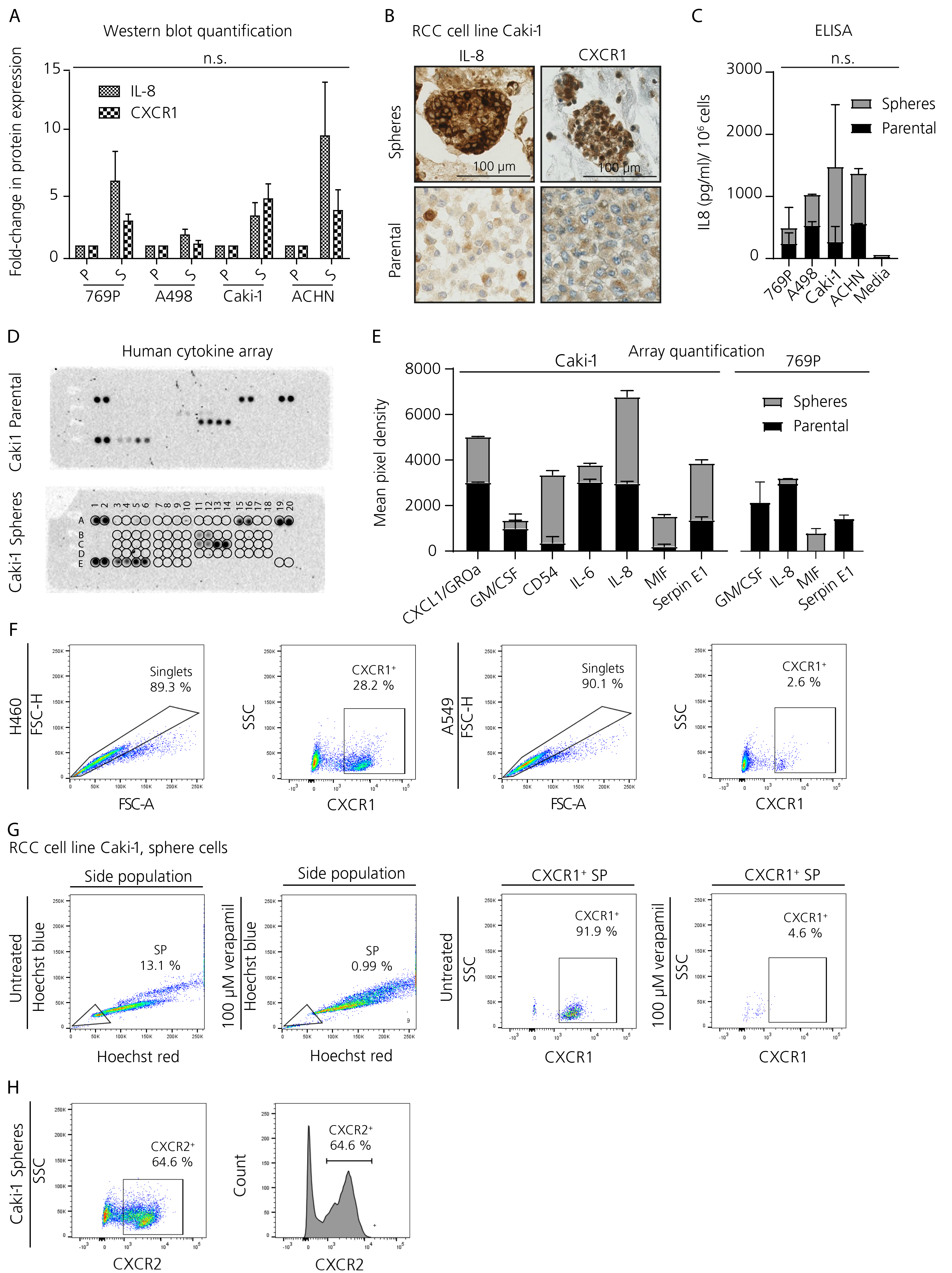

Supplement: Supplementary file 4 — Figure S2. Side population cells are characterized by CXCR1 expression [file PATH-248-377-s002.tif]

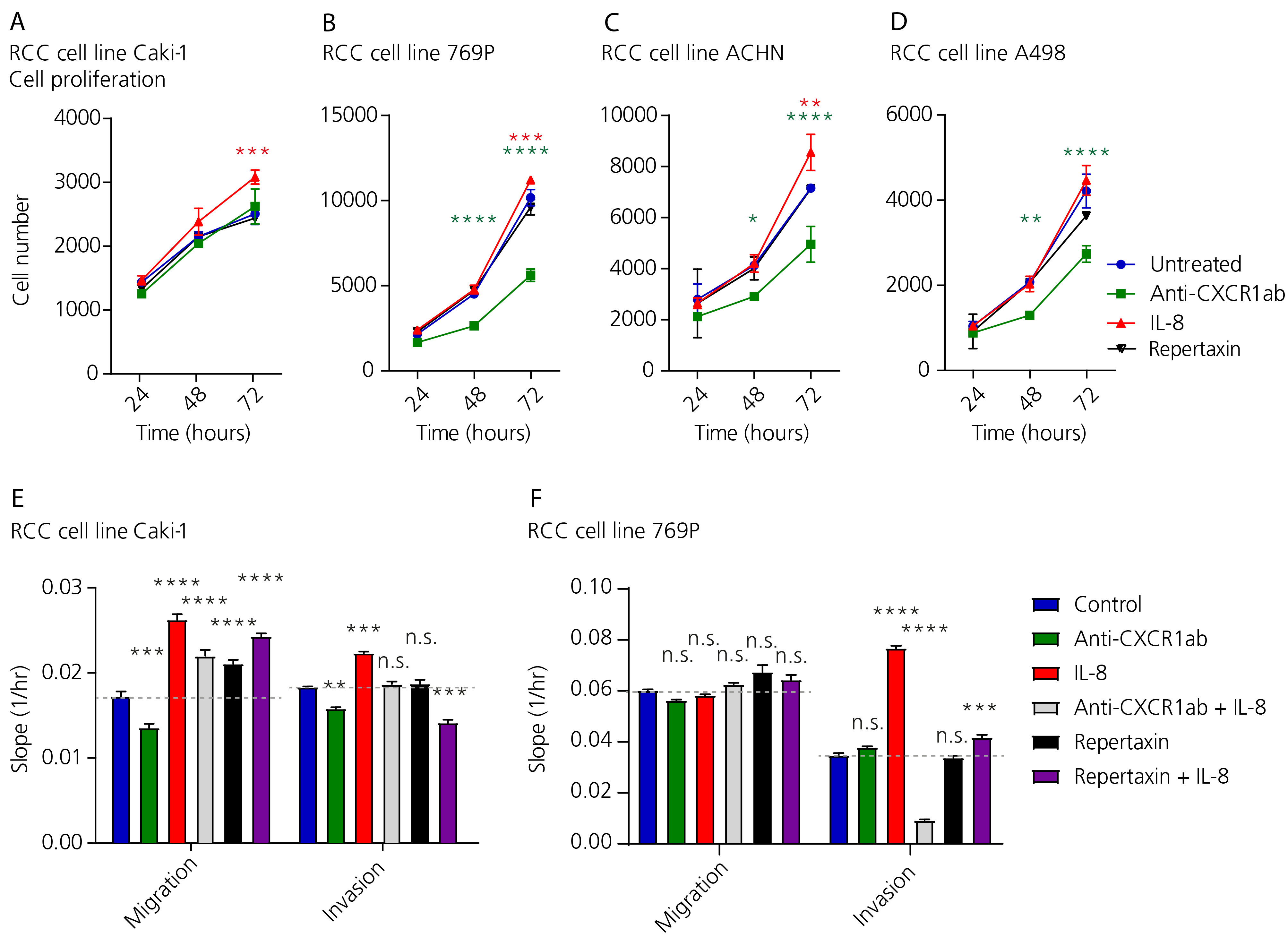

Supplement: Supplementary file 5 — Figure S3. IL‐8/CXCR1 signaling affects cell proliferation, migration and invasion of ccRCC cell lines [file PATH-248-377-s003.tif]

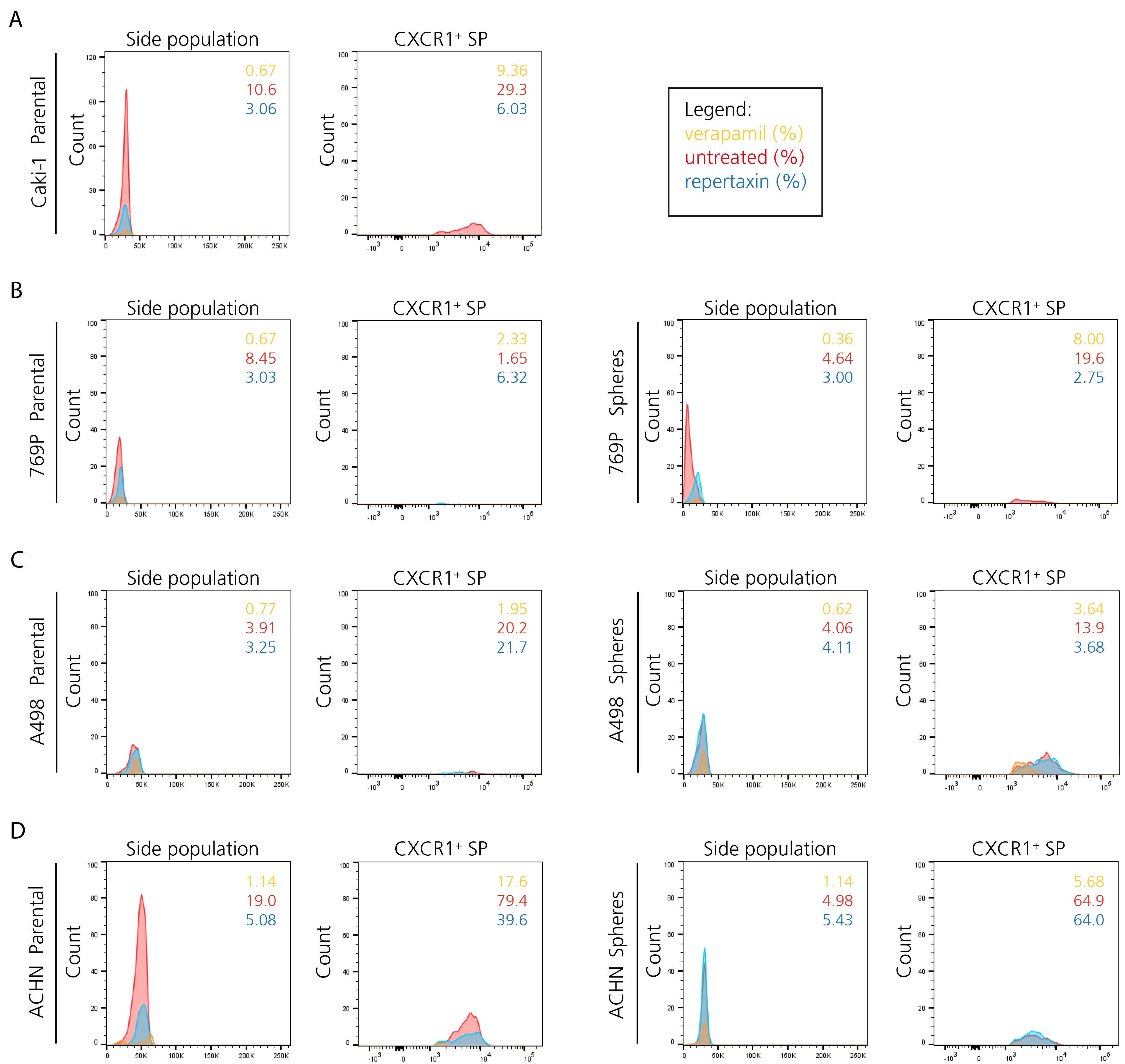

Supplement: Supplementary file 6 — Figure S4. Repertaxin treatment reduced SP and CXCR1+ cells [file PATH-248-377-s004.tif]

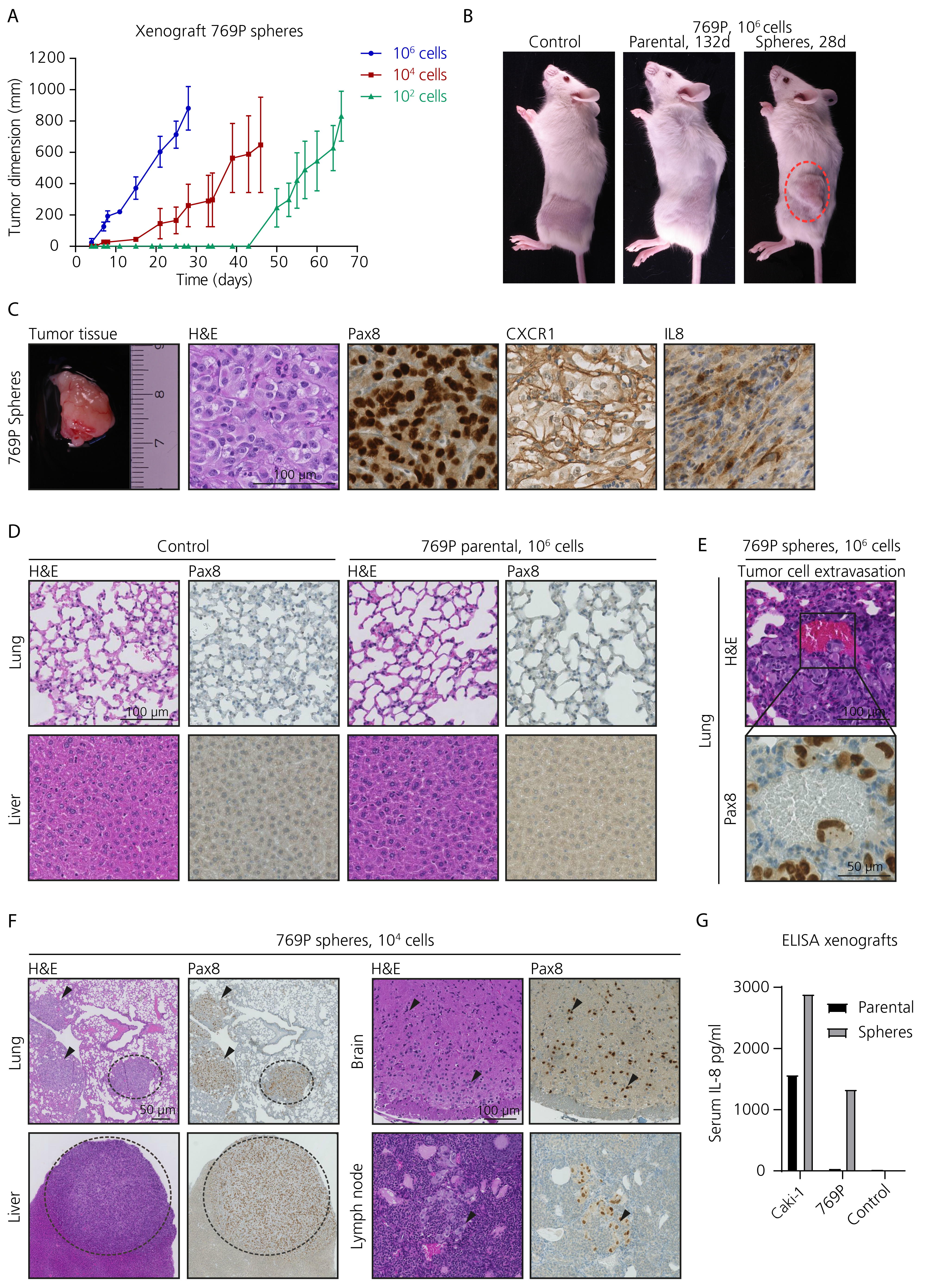

Supplement: Supplementary file 7 — Figure S5. Tumor xenografts derived from the 769P cell line [file PATH-248-377-s005.tif]
