# Supplementary material for: IL‐8 and CXCR1 expression is associated with cancer stem cell‐like properties of clear cell renal cancer
Source: J Pathol. 2019 Apr 11;248(3):377–89. doi: 10.1002/path.5267 (PMC6618115; doi:10.1002/path.5267)
Supplement: Supplementary file 8 — Table S1. Detailed antibody information Table S2. Human cytokine array panel Table S3. Flow cytometry data for SP and CXCR1/2+ cell populations in parental and sphere cells in ccRCC cell lines Table S4. Flow cytometry data for SP and CXCR1+ cells in parental and spheres before and after repertaxin treatment [file PATH-248-377-s008.docx]

**IL-8 and CXCR1 expression is associated with cancer stem cell-like properties of clear cell renal cancer**

Corrò C *et al*. *J Pathol* DOI: 10.1002/path.5267

**Supplementary tables S1-S5**

**Table S1.** Detailed antibody information.

| **Immunohistochemistry** | | | | | | | | | | |
| --- | --- | --- | --- | --- | --- | --- | --- | --- | --- | --- |
| **Antibody against** | **Name/**  **clone** | | **Dilution** | | **Supplier** | | **IHC system** | | **Reagents** | **Protocol/**  **detection** |
| PanCKa | LU-5 | | 1:50 | | DAKO A/S (Glostrup, Denmark) | | Ventana Benchmark Ultra (Roche Diagnostics, Basel, Switzerland) | | OptiView Kit (Roche Diagnostics) | Pretreatment: Protease1  4 min |
| CK7 | OV-TL 12/30 | | 1:100 | | DAKO A/S | | Ventana Benchmark Ultra | | OptiView Kit | Pretreatment: Protease1  4 min |
| PAX8 | Paired box 8 | | 1:400 | | Protein Tech Group, Inc. (Rosemont, IL, USA) | | Ventana Benchmark Ultra | | OptiView Kit | Pretreatment: Tris-EDTA-Borate Buffer  80 min |
| CAIX | Carbonic Anhydrase IX polyclonal | | 1:6000 | | Abcam (Cambridge, UK) | | Leica Bond (Leica Biosystems, Wetzlar, Germany) | | Bond Polymer Refine Kit (Leica Biosystems) | Pretreatment: Tris-EDTA- Borate Buffer  30 min |
| Vimentin | Vim3B4 | | 1:800 | | Abcam | | Leica Bond | | Bond Polymer Refine Kit | Pretreatment: Tris-EDTA- Borate Buffer  60 min |
| E-cadherin | EP700Y | | 1:200 | | CellMarque (Rocklin, CA, USA) | | Ventana Benchmark Ultra | | OptiView Kit | Pretreatment: Tris-EDTA- Borate Buffer  40 min |
| Snail/Slug |  | | 1:200 | | Abcam | | Ventana Discovery Ultra (Roche Diagnostics) | | ChromoMap Kit (Roche Diagnostics) | Pretreatment: Tris-EDTA- Borate Buffer  30 min |
| CXCR1 | 42705 | | 1:2000 | | R&D Systems (Minnea-polis, MN, USA) | | Ventana Discovery Ultra | | ChromoMap Kit | Pretreatment: Protease1  4 min |
| IL-8 | 6217 | | 1:100 | | R&D Systems | | Leica Bond | | Bond Polymer Refine Kit | Pretreatment: Tris-EDTA- Borate Buffer  90 min |
| CD105 | 3A9 | | 1:400 | | Novus Biologicals (Centennial CO, USA) | | Ventana Benchmark Ultra | | OptiView Kit | Pretreatment: Tris-EDTA- Borate Buffer  60 min |
| **Mass cytometry** | | | | | | | | | | |
| **Antibody against** | | **Name/**  **clone** | | **Dilution** | | **Supplier** | |  | | |
| CD13 | | WM15 | | 2 µg/ml | | Biolegend (San Diego, CA, USA) | |  |  |  |
| CD24 | | 32D12 | | 2 µg/ml | | Miltenyi Biotec. (Bergisch Gladbach, Germany) | |  |  |  |
| CD44 | | IM7 | | 0.5 µg/ml | | Becton Dickinson (Allschwil, Switzerland) | |  |  |  |
| CD10 | | HI10a | | 2 µg/ml | | Biolegend | |  | | |
| CXCR4 | | 12G5 | | 4 µg/ml | | Biolegend | |  |  |  |
| Vimentin | | RV202 | | 0.1 µg/ml | | Becton Dickinson | |  |  |  |
| E-Cadherin | | 36/E-Cadherin | | 2 µg/ml | | Becton Dickinson | |  |  |  |

**Table S2.** Human cytokine array panel.

| **Coordinate** | **Target/Control** |
| --- | --- |
| A1, A2 | Reference Spot |
| A3, A4 | Complement Component 5/5a |
| A5, A6 | CD40 Ligand |
| A7, A8 | G-CSF |
| A9, A10 | GM-CSF |
| A11, A12 | GROα |
| A13, A14 | I-309 |
| A15, A16 | CD54 |
| A17, A18 | IFN-γ Type II |
| A19, A20 | Reference Spot |
| B3, B4 | IL-1α |
| B5, B6 | IL-1β |
| B7, B8 | IL-1ra |
| B9, B10 | IL-2 |
| B11, B12 | IL-4 |
| B13, B14 | IL-5 |
| B15, B16 | IL-6 |
| B17, B18 | IL-8 |
| C3, C4 | IL-10 |
| C5, C6 | IL-12 |
| C7, C8 | IL-13 |
| C9, C10 | IL-16 |
| C11, C12 | IL-17 |
| C13, C14 | IL-17E |
| C15, C16 | IL-23 |
| C17, C18 | IL-27 |
| D3, D4 | IL-32α |
| D5, D6 | CXCL10 |
| D7, D8 | CXCL11 |
| D9, D10 | CCL2 |
| D11, D12 | MIF |
| D13, D14 | MIP-1α |
| D15, D16 | MIP-1β |
| D17, D18 | Serpin E1 |
| E1, E2 | Reference Spot |
| E3, E4 | RANTES CCL5 |
| E5, E6 | SDF-1 CXCL12 |
| E7, E8 | TNF-α |
| E9, E10 | sTREM-1 |
| E19, E20 | Negative Control |

**Table S3.** Flow cytometry data for SP and CXCR1/2^+^ cell populations in parental and sphere cells in ccRCC cell lines (n>2).

|  | **769P** | | **A498** | | **Caki-1** | | **ACHN** | |
| --- | --- | --- | --- | --- | --- | --- | --- | --- |
|  | parental | spheres | parental | spheres | parental | spheres | parental | spheres |
| **SP** | 3.8±0.6% | 14.6±1.8% | 3.5±1.9% | 14.8±2.9% | 6±0.68% | 15.3±3.8% | 2.1±0.7% | 13.6±7.6% |
| **CXCR1^+^ cells** | 4.7±0.6% | 9.1±3.3% | 8.2±0.7% | 29.9±5% | 6.6±0.9% | 15±0.6% | 8±0.2% | 14.7±6% |
| **CXCR1^+^ SP** | 3.7±1.1% | 23.1±1% | 14.6±5.7% | 93.7±8.9% | 20.2±8.8% | 71.3±4.1% | 7.5±4.7% | 46.5±9.5% |
| **CXCR2^+^ cells** | n.a. | 72±1.48% | n.a. | n.a. | n.a. | 64.7±0.2% | n.a. | 63±5.2% |

n.a. = Not assessed

**Table S4. Flow cytometry data for SP and CXCR1^+^ cells in parental and spheres before and after repertaxin treatment.**

|  | **769P** | | **A498** | | **Caki-1** | | **ACHN** | |
| --- | --- | --- | --- | --- | --- | --- | --- | --- |
|  | Parental | Spheres | Parental | Spheres | Parental | Spheres | Parental | Spheres |
| **SP** | 8.45% | 4.64% | 3.91% | 4.06% | 10.6% | 11.4% | 19% | 4.98% |
| **CXCR1^+^ cells** | 31.6% | 20.5% | 4.19% | 6.06% | 23.2% | 27.0% | 16.9% | 18.2% |
| **CXCR1^+^ SP** | 1.6% | 19.6% | 20.2% | 13.9% | 29.3% | 85.0% | 24.9% | 64.4% |
| **+ Repertaxin** | | | | | | | | |
| **SP** | 3.03% | 3.04% | 3.25% | 4.11% | 3.06% | 7.3% | 5.8% | 5.43% |
| **CXCR1^+^ cells** | 26.5% | 8.9% | 3.26% | 5.15% | 20.0% | 15.5% | 31.5% | 26.2% |
| **CXCR1^+^ SP** | 6.32% | 2.75% | 21.7% | 3.7% | 6.03% | 44.0% | 79.4% | 64.0% |

**Table S5.** Patients’ details for TMA and RNA analyses.

This table is provided as an Excel file ‘Table S5.xlsx’.
